# Assessing Lymph Node Metastasis Risk in Patients With Mucosal Gastric Cancer: A Comparison of the WHO and Japanese Criteria and Implications for Endoscopic Submucosal Dissection

**DOI:** 10.1002/jgh3.70432

**Published:** 2026-06-19

**Authors:** Jane Chungyoon Kim, Yo‐Seok Cho, Yoonjin Kwak, Seong‐Ho Kong, Do Joong Park, Soo‐Jeong Cho, Hyuk‐Joon Lee, Hye Seung Lee, Han‐Kwang Yang

**Affiliations:** ^1^ Department of Surgery Seoul National University Hospital Seoul South Korea; ^2^ Department of Pathology Seoul National University Hospital Seoul South Korea; ^3^ Department of Surgery Seoul National University College of Medicine Seoul South Korea; ^4^ Cancer Research Institute, Seoul National University College of Medicine Seoul South Korea; ^5^ Department of Internal Medicine Seoul National University College of Medicine Seoul Korea; ^6^ Department of Internal Medicine Liver Research Institute, Seoul National University College of Medicine Seoul Korea

**Keywords:** early gastric cancer, endoscopic submucosal dissection, lymph node metastasis, risk factors, undifferentiated type

## Abstract

**Background and Aim:**

Endoscopic submucosal dissection (ESD) is an established treatment for early gastric cancer. However, concerns remain regarding the risk of lymph node metastasis (LNM) under expanded criteria and its applicability across different populations. This study assessed LNM risk in patients with mucosal gastric cancer using WHO and Japanese criteria.

**Methods:**

This retrospective cohort study included 2232 patients with mucosal gastric cancer who underwent curative gastrectomy. Clinicopathological factors, including tumor size, histological type, ulceration, and lymphovascular invasion (LVI), were analyzed. Independent risk factors for LNM were identified through multivariate logistic regression.

**Results:**

LNM occurred in 3.5% of the patients. Undifferentiated‐type (UD‐type) tumors within the updated ESD criteria (size ≤ 2 cm, without ulceration) had a 2.1% LNM occurrence rate. In contrast, differentiated‐type (D‐type) tumors (no ulceration with size > 2 cm or presence of ulceration with size ≤ 3 cm) showed 3.3% and 1.4% LNM occurrence rates, respectively. The pattern of lymph node metastasis at each station indicated that D1+ lymph node dissection is sufficient for mucosal gastric cancers. Multivariate analysis identified large tumor size, ulceration, UD‐type, and LVI as significant LNM risk factors, with UD‐type tumors consistently showing higher LNM rates than D‐type tumors across different size categories.

**Conclusions:**

These findings underscore the LNM risk in mucosal gastric cancer under the updated ESD criteria. Individualized treatment and counseling, particularly for young patients with good performance status, are essential. A cautious approach aligning treatment with patient characteristics is recommended.

## 1 Introduction

Gastric cancer remains a significant global health challenge; it is the fifth most common cancer worldwide, and over 60% of gastric cancer cases occur in Eastern Asia [1]. Eastern Asian nations have made considerable progress in early detection through robust screening programs. Notably, Japan and Korea report that over 60% of gastric cancer diagnoses occur at early stages [2, 3, 4].

Curative gastrectomy has been the primary treatments for early gastric cancer (EGC). However, there has been a concerted effort to develop endoscopic techniques such as endoscopic mucosal resection (EMR) and endoscopic submucosal dissection (ESD) [5]. The development of ESD has enabled resection of mucosal lesions, regardless of size or the presence of ulcers [6]. Previously, Japanese guidelines for ESD restricted patient selection to specific criteria, including lesions less than 2 cm, differentiated‐type mucosal cancers, and the absence of ulcerative findings [7]. However, pivotal studies conducted in Japan have significantly altered these guidelines. Large cohort studies of Japanese patients who underwent surgery revealed a 0% lymph node metastasis (LNM) rate in cases of large‐sized, differentiated mucosal tumors, as well as small‐sized, ulcer‐negative, undifferentiated mucosal tumors [8, 9].

Consequently, the current Japanese guidelines now absolutely recommend ESD for cases that meet expanded criteria: (1) differentiated‐type mucosal cancer without ulcers, regardless of size; (2) differentiated‐type mucosal cancer less than 3 cm, irrespective of ulcer presence; and (3) undifferentiated‐type mucosal cancer less than 2 cm without ulcers [10]. Recently, the Korean Practice Guidelines for Gastric Cancer were updated to endorse ESD for large‐sized, differentiated‐type mucosal cancers and cautiously recommended considering endoscopic resection for patients with undifferentiated mucosal tumors less than 2 cm and without ulcers [11].

In contrast, several studies have indicated a non‐negligible risk of LNM in patients with EGC. We previously reported a retrospective study of a patient with mucosal gastric cancer undergoing gastrectomy, showing a 2.3% LNM rate in undifferentiated‐type EGC meeting the extended ESD criteria [12]. Notably, several studies of Chinese patients with mucosal gastric cancer within the ESD criteria, who underwent curative gastrectomy, revealed a 4.1% to 5.0% LNM risk [13, 14]. A national data analysis from the United States also showed a 20% LNM risk in patients with even differentiated‐type mucosal gastric cancer [15].

The differences in LNM risk between Japanese patients with mucosal gastric cancer and those from other regions may be attributed to variations in pathological diagnostic practices, including specimen section intervals. The Japanese Gastric Cancer Association (JGCA) permits EGC diagnosis based on severe cytological atypia, which may result in a higher rate of mucosal cancer diagnoses compared to the WHO criteria, where cancer depth is assessed through direct structural invasion [16, 17]. In Japan, lesions classified as high‐grade dysplasia in other regions may be diagnosed as mucosal gastric cancer [18, 19]. These differences in diagnostic standards highlight the importance of careful consideration when applying the expanded ESD criteria to patients diagnosed according to WHO guidelines. In this study, we aimed to evaluate the LNM risk in a large cohort of patients with mucosal gastric cancer. The primary objective of this study is to assess the absolute LNM risk across classic and expanded ESD subgroups based on WHO diagnostic criteria. The secondary objectives are to identify clinicopathologic risk factors for LNM and to evaluate the distribution of metastatic lymph node stations to assess the adequacy of D1+ lymph node dissection.

## 2 Materials and Methods

### 2.1 Patients

In this retrospective cohort study, we included patients with mucosal gastric cancer who underwent curative gastrectomy with lymphadenectomy at Seoul National University Hospital (SNUH) between 2013 and 2022. All patients were pathologically confirmed to be pT1a following surgery. At our institution, gastrectomy is performed for all undifferentiated‐type gastric adenocarcinomas, regardless of tumor size or ulceration. Gastrectomy and LND procedures were performed in accordance with the Korean Gastric Cancer Association guidelines [11]. For clinical T1 patients, we chose distal, total, pylorus‐preserving, or proximal gastrectomy according to the tumor's location or extent and performed either D1 or D1+ LND. We excluded patients with more than one synchronous gastric cancer within the resected stomach specimen, as well as those clinically suspected of having LNM during preoperative workups such as endoscopic ultrasound or computed tomography. We collected demographic data (including age at diagnosis and sex), clinical information (cancer location, tumor size, presence of ulcer, surgery type, and approach), and pathological data (histologic type [WHO], Lauren classification, lymphovascular invasion [LVI], tumor depth, number of metastatic lymph nodes, lymph node stations with metastasis, and number of resected lymph nodes). Ulcer presence was defined as either ulcerative lesions or ulceration scars in the tumor, identified through preoperative gastroscopy and examination of the surgical specimen. This definition is consistent with the Japanese ESD guidelines, in which both active ulceration and ulcer scars are considered ulcer‐positive. Because ulceration is not always explicitly reported in endoscopic descriptions, whereas it is systematically assessed in pathological examination, histopathological findings were used as the primary reference for ulcer status. When discrepancies occurred, ulcer status was primarily adjudicated based on pathological findings.

### 2.2 Histopathological Evaluation

Experienced pathologists at SNUH, specialized in gastric cancer, conducted the pathologic review, following the standardized report established by the Korean Society of Pathologists for gastric cancer [11, 20, 21]. After gastrectomy, surgeons classified and labeled the lymph nodes into different stations before sending the specimen to the pathologists. Stomach specimens were immediately fixed in formalin, and after gross inspection, they were prepared into paraffin blocks by sectioning at 4 mm intervals. Tumor depth was determined according to the American Joint Committee on Cancer (AJCC, 8th edition) [22]. According to AJCC guidelines, the presence of LVI was recorded separately from the depth of invasion [22]. Histological type was determined based on WHO guidelines [17], with diagnosis based on the dominant component of the tumor. For mixed‐type tumors, the histological type occupying the majority was described first, followed by other types, with the proportions of each histological type indicated. Tumors were classified as differentiated‐type (D‐type) or undifferentiated‐type (UD‐type) adenocarcinoma according to the Japanese pathologic guidelines used in the current ESD criteria [10, 16]. D‐type includes well‐differentiated adenocarcinoma, moderately differentiated adenocarcinoma, and papillary carcinoma, while UD‐type includes poorly differentiated adenocarcinoma, poorly cohesive carcinoma, and signet ring cell carcinoma. Tumors exhibiting both D‐ and UD‐type characteristics were categorized based on the majority component. The total number of harvested lymph nodes and the number of lymph nodes involved with metastasis were recorded, with lymph nodes labeled by station by the surgeons.

### 2.3 Statistical Analysis

Continuous variables are presented as mean ± standard deviation and were compared using the Student's *t*‐test. Categorical variables are expressed as numbers (percentages) and were compared using the Chi‐square test or Fisher's exact test. Univariate and multivariate analyses of clinicopathological factors for LNM were performed using binary logistic regression to identify risk factors for LNM. Odds ratios, 95% confidence intervals, and *p*‐values were calculated. Statistical analysis was performed using either SPSS Statistics version 29 or STATA version 18.0.

## 3 Results

### 3.1 Patient Characteristics

We included 2232 patients with mucosal gastric cancer who underwent gastrectomy. Of these patients, 79 (3.5%) showed LNM. The patients were categorized into LNM (*n* = 79) and non‐LNM (*n* = 2153) groups. Table 1 details their clinicopathological differences and the univariate analysis of potential risk factors for LNM. No significant age differences were observed between the groups. The LNM group had a higher proportion of female patients (*n* = 48; 4.6%) than male patients (*n* = 31; 2.6%). In contrast, the non‐LNM group had more male patients (*n* = 1152; 97.4%) than female patients (*n* = 1001; 95.4%) (*p* = 0.013). Tumor size was larger in the LNM group (3.41 ± 2.01 cm) compared to the non‐LNM group (2.53 ± 1.59 cm) (*p* < 0.001). LNM was significantly higher in the presence of ulceration (*n* = 18; 5.9%) than in its absence (*n* = 61; 3.2%) (*p* = 0.018). Patients with UD‐type histology had significantly higher rates of LNM (*n* = 58; 4.3%) than those with D‐type histology (*n* = 21; 2.4%) (*p* = 0.017). LVI was very high in the LNM group (*n* = 13; 26.0%), and the absence of LVI showed significantly lower LNM rates (*n* = 66; 3.0%) (*p* < 0.001). Lauren classification revealed a significantly higher proportion of diffuse and mixed‐type tumors in the LNM group (Intestinal [*n* = 19; 2.2%], Diffuse [*n* = 44; 3.7%], and Mixed [*n* = 15; 10.1%]) (*p* < 0.001). Lastly, the LNM group had a higher average of harvested lymph nodes (41.63 ± 15.19) than the non‐LNM group (37.67 ± 14.69) (*p* = 0.019). However, gastric cancer location, both longitudinal and cross‐sectional, was not a significant factor for LNM.

**TABLE 1:** Clinicopathological risk factors for lymph node metastasis in mucosal gastric cancer patients.

	LNM (+) (*N* = 79)	LNM (−) (*N* = 2153)	*p*
Age, mean ± std. (years)	58.10 ± 11.69	59.32 ± 11.74	0.364
Sex, *n* (%)			
Male	31 (2.6%)	1152 (97.4%)	0.013
Female	48 (4.6%)	1001 (95.4%)	—
Tumor size, mean ± std. (cm)	3.41 ± 2.01	2.53 ± 1.59	< 0.001
Tumor size range, *n* (%)			
≤ 2 cm	22 (2.1%)	1038 (97.9%)	< 0.001
> 2 cm	57 (4.9%)	1115 (95.1%)	—
Ulceration, *n* (%)			
Present	18 (5.9%)	289 (94.1%)	0.018
Absent	61 (3.2%)	1864 (96.8%)	—
Longitudinal location, *n* (%)			0.225
Upper 1/3	5 (2.0%)	243 (98.0%)	
Middle 1/3	31 (3.4%)	872 (96.6%)	
Lower 1/3	48 (4.2%)	1094 (95.8%)	
Cross‐sectional location, *n* (%)			0.254
Anterior wall	19 (3.7%)	491 (96.3%)	
Posterior wall	20 (3.3%)	580 (96.7%)	
Lesser curvature	26 (3.1%)	814 (96.9%)	
Greater curvature	24 (5.2%)	436 (94.8%)	
Histology, *n* (%)			
Differentiated	21 (2.4%)	861 (97.6%)	0.017
Undifferentiated	58 (4.3%)	1292 (95.7%)	
Lymphovascular invasion, *n* (%)			< 0.001
Present	13 (26.0%)	37 (74.0%)	
Absent	66 (3.0%)	2116 (97.0%)	
Lauren classification, *n* (%)			< 0.001
Intestinal	19 (2.2%)	862 (97.8%)	
Diffuse	44 (3.7%)	1149 (96.3%)	
Mixed	16 (10.1%)	142 (89.9%)	
Number of LNs			
Metastatic, mean ± std	3.20 ± 3.88	0	< 0.001
Harvested, mean ± std	41.63 ± 15.19	37.67 ± 14.69	0.019

Abbreviations: LNM, lymph node metastasis; std., standard deviation.

### 3.2 LNM Rates of Patients Within the ESD Criteria

**FIGURE 1:**
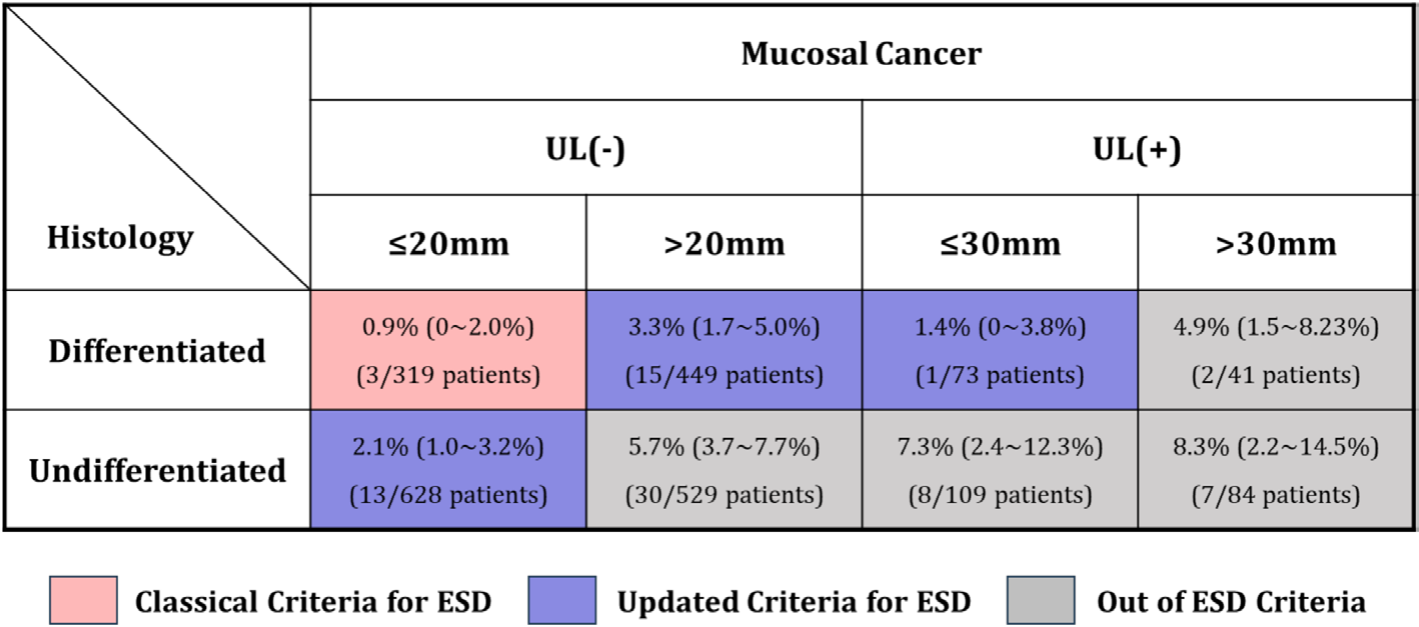
Lymph node metastasis rates of patients within the endoscopic submucosal dissection criteria.

The calculated LNM rates for patients who fit the updated ESD criteria are shown in Figure 1. The traditional ESD criteria, which include D‐type tumors with no ulcer and size less than or equal to 2 cm, showed a 0.9% (95% CI: 0%–2.0%) LNM rate. However, the revised ESD criteria showed the following LNM rates: 1) D‐type without ulcer but size more than 2 cm had a 3.3% LNM rate (95% CI: 1.7%–5.0%), 2) D‐type with ulcer and size less than 3 cm had a 1.4% LNM rate (95% CI: 0%–3.8%), 3 UD‐type without ulcer and size less than 2 cm had a 2.1% LNM rate (95% CI: 1.0%–3.2%). Mucosal tumors that did not meet the ESD criteria had much higher LNM rates, ranging from 4.9%–8.3%. These LNM rates represent occult metastasis identified in a surgically treated cohort and should be interpreted with this context when extrapolating to patients treated with ESD.

We conducted a comprehensive examination of the histopathological characteristics of mucosal cancers accompanied by LNM. Additionally, we incorporated the absence of LVI as a key criterion. Our study identified subgroups of patients within each ESD criterion, which are presented in Table 2. The first subgroup comprised 11 patients with undifferentiated mucosal tumors measuring less than or equal to 2 cm, characterized by the absence of ulcers and LVI. Of these 11 patients, four exhibited mixed‐type tumors displaying a combination of different histological types. Furthermore, we identified 12 patients with D‐type histology within the updated ESD criteria and without LVI, and five of these had mixed‐type tumors containing both moderately differentiated adenocarcinoma (TUB2) and poorly cohesive carcinoma, NOS (POR2), or signet ring cell components.

**TABLE 2:** Histopathological details of the patients within the endoscopic submucosal dissection criteria.

Patient	Differentiation	Size (cm)	Ulcer	LVI	Histology	No. metastatic LN
1	UD‐type	1.2	(−)	(−)	POR2	1
2	UD‐type	1.3	(−)	(−)	POR2 (60%) + SRC (20%) + POR1 (20%)	1
3	UD‐type	1.3	(−)	(−)	POR2	1
4	UD‐type	1.4	(−)	(−)	POR1 (60%) + SRC (40%)	7
5	UD‐type	1.4	(−)	(−)	SRC	4
6	UD‐type	1.6	(−)	(−)	POR2	2
7	UD‐type	1.6	(−)	(−)	POR1	1
8	UD‐type	1.6	(−)	(−)	SRC	3
9	UD‐type	1.6	(−)	(−)	POR1 with SRC component	3
10	UD‐type	2.0	(−)	(−)	POR1 (60%) + SRC (40%)	1
11	UD‐type	2.0	(−)	(−)	SRC	2
12	D‐type	1.2	(−)	(−)	TUB1	1
13	D‐type	2	(−)	(−)	TUB2	1
14	D‐type	2.2	(+)	(−)	TUB2	1
15	D‐type	2.4	(−)	(−)	TUB2	2
16	D‐type	2.8	(−)	(−)	TUB2	1
17	D‐type	3.2	(−)	(−)	TUB2 (60%) + SRC (40%)	1
18	D‐type	3.3	(−)	(−)	TUB2 with SRC component	1
19	D‐type	4.5	(−)	(−)	TUB1	1
20	D‐type	5.5	(−)	(−)	TUB2 (80%) + SRC (20%)	1
21	D‐type	5.6	(−)	(−)	TUB2 (60%) + SRC (40%)	8
22	D‐type	6.7	(−)	(−)	TUB2	1
23	D‐type	10.8	(−)	(−)	TUB1 (90%) + POR2 (10%)	1

Abbreviations: D‐type, differentiated‐type; LN, lymph node; LNM #, number of lymph node metastasis; POR1, tubular, poorly‐differentiated; POR2, poorly cohesive, NOS; SRC, Poorly cohesive, signet ring cell type; TUB1, tubular, well‐differentiated; TUB2, tubular, moderately differentiated; UD‐type, undifferentiated‐type.

**FIGURE 2:**
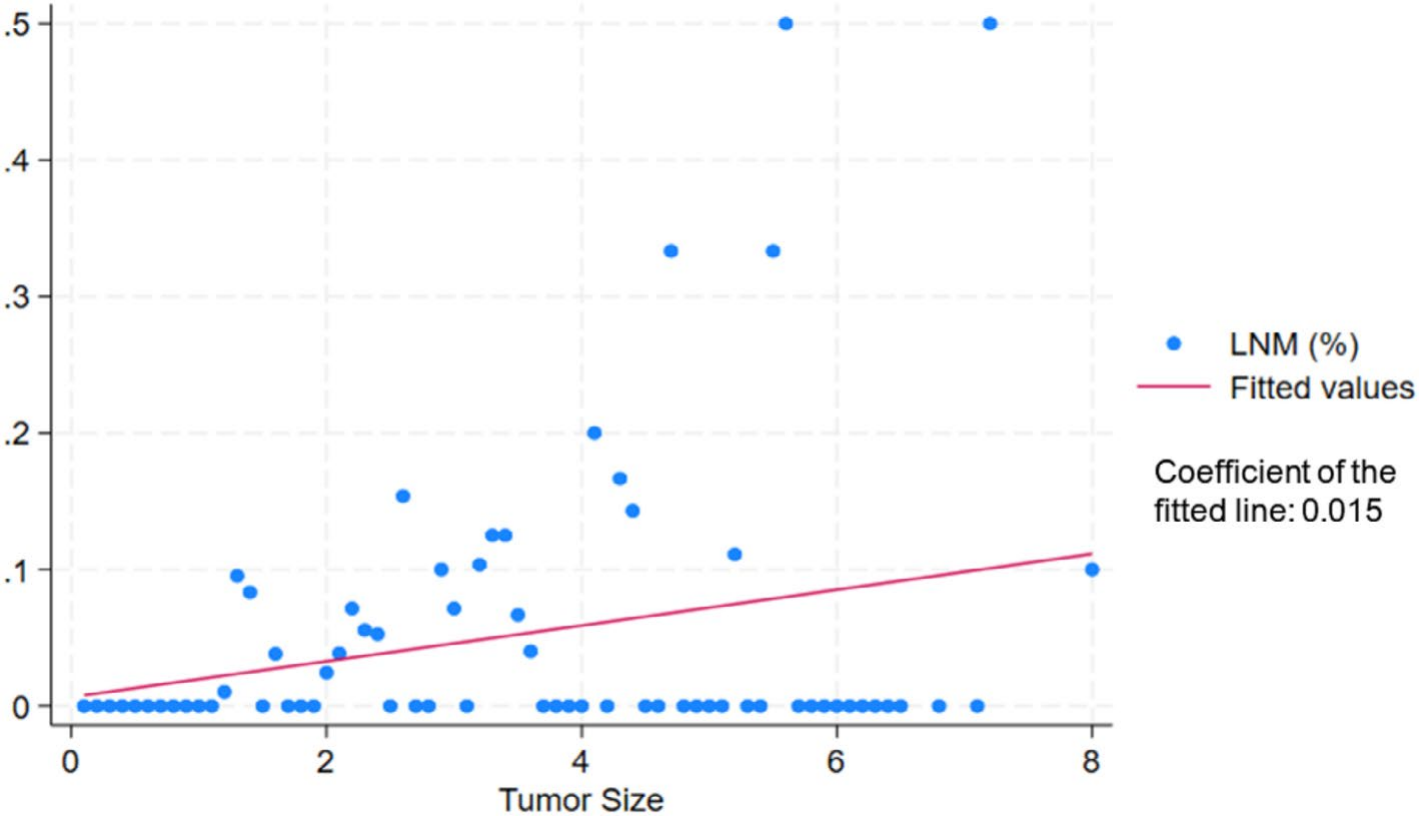
Scatterplot of lymph node metastasis rate versus tumor size for undifferentiated‐type mucosal tumors without ulcer and lymphovascular invasion.

We used a scatterplot with linear fitting to descriptively visualize the trend in observed LNM frequency among tumors meeting the ESD criteria (see Figure 2). The fitted line illustrates an increasing tendency in the observed proportion of LNM with increasing tumor size in UD‐type mucosal cancers without ulceration or LVI. The slope of the fitted line was positive (0.015), reflecting this upward trend. Notably, at the 2 cm size threshold—corresponding to the current expanded ESD cutoff for UD‐type tumors—the fitted line suggests a LNM frequency of approximately 3%.

### 3.3 Lymph Node Stations Where Metastasis Was Observed

**FIGURE 3:**
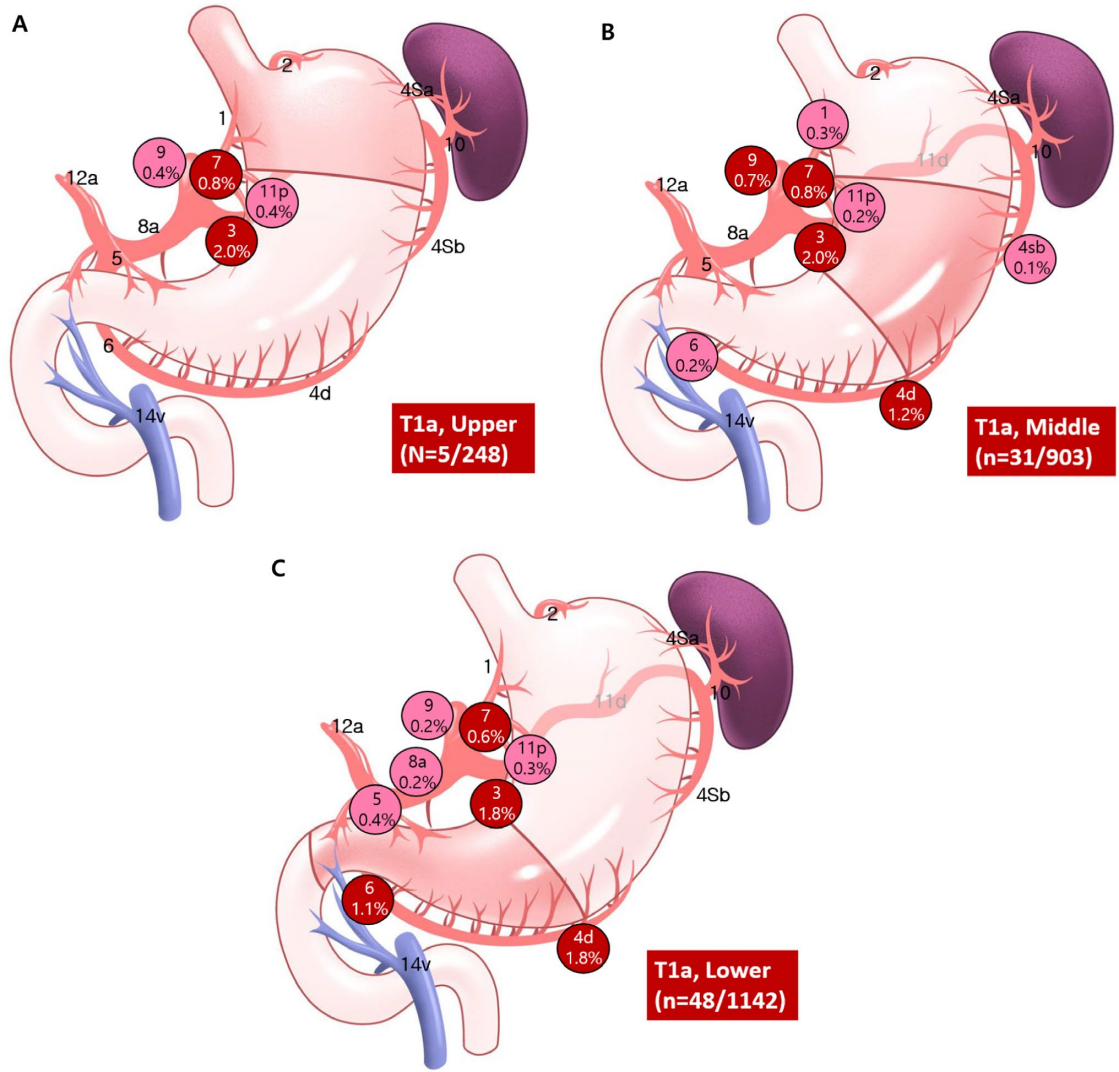
Regional lymph node metastasis rates in patients with mucosal gastric cancer, stations 1–12. (A) Overall lymph node metastasis rates in T1a upper gastric cancer. (B) Overall lymph node metastasis rates in T1a middle gastric cancer. (C) Overall lymph node metastasis rates in T1a lower gastric cancer.

The results also revealed significant amounts of LNM in patients with mucosal gastric cancer. We investigated the number of metastases at each lymph node station according to the longitudinal tumor location to delineate the patterns of lymph node stations where metastasis occurred. Of the 248 patients with mucosal cancer located in the upper stomach, five had LNM. The most common site of metastasis was LN #3, with metastasis also observed in LN #7, 9, and 11p (Figure 3A). For cancer located in the middle stomach, 31 out of 903 patients had LNM. The most common sites of metastasis were lymph nodes #3, 4d, 7, 8, and 9 (Figure 3B). Lastly, 48 out of 1142 patients with lower gastric cancer had LNM. The most common sites of metastasis were LN #3, 4d, 6, and 7 (Figure 3C). No LNM was observed in LN stations #2, 4sa, 10, 11d, and 12a. Only the lymph node stations covered by D1 + 11p lymph node dissection showed risks of metastasis.

### 3.4 Multivariate Analysis of Risk Factors for LNM in EGC

A multivariate analysis was conducted to identify potential risk factors for LNM in patients with EGC, and the results are summarized in Table 3. Similar to the univariate analysis, the multivariate analysis showed age as a non‐significant determinant for LNM. For patients older than 65 years, the OR for LNM was 1.152 (95% CI: 0.685–1.936, *p* = 0.594) compared to those less than 65 years old. Sex was significant in the univariate analysis; however, its significance was not retained in the multivariate analysis. Female patients had an OR of 1.518 (95% CI: 0.933–2.469, *p* = 0.093) for LNM. The prominent risk factors in the multivariate analysis included tumor size, presence of ulcers, undifferentiated histology, and LVI. Tumors greater than or equal to 2 cm were associated with higher odds of LNM, with an OR of 2.180 (95% CI: 1.302–3.648, *p* = 0.003) compared to tumors less than 2 cm. The presence of an ulcer had an OR of 1.797 (95% CI: 1.019–3.170, *p* = 0.043), while undifferentiated histology also significantly contributed to LNM, with an OR of 2.068 (95% CI: 1.172–3.648, *p* = 0.012). Finally, LVI emerged as a substantial risk factor with an OR of 9.966 (95% CI: 4.859–20.440, *p* < 0.001).

**TABLE 3:** Multivariate analysis of lymph node metastasis risk factors.

	Multivariate analysis	
	OR (95% CI)	*p*
Age (years)		
< 65	1	—
≥ 65	1.152 (0.685–1.936)	0.594
Sex		
Male	1	—
Female	1.518 (0.933–2.469)	0.093
Tumor size (cm)		
≤ 2	1	—
> 2	2.180 (1.302–3.648)	0.003
Ulceration		
Absent	1	—
Present	1.797 (1.019–3.170)	0.043
Histology		
Differentiated	1	—
Undifferentiated	2.068 (1.172–3.648)	0.012
Lymphovascular invasion		
Absent	1	—
Present	9.966 (4.859–20.440)	< 0.001

### 3.5 LNM Rates According to Risk Factors

We explored the LNM rates in relation to specific risk factors for mucosal tumors (Table 4). UD‐type tumors consistently demonstrated higher LNM rates than their D‐type counterparts. For tumors measuring less than or equal to 2 cm, the metastasis rate was 2.7% for UD‐type and 0.8% for D‐type. For medium‐sized tumors (ranging from 2–3 cm), D‐type tumors showed a metastasis rate of 3.0%, while UD‐type tumors recorded a rate of 5.1%. For larger tumors (greater than 3 cm), the metastasis rates were 3.8% for D‐type and 6.6% for UD‐type.

**TABLE 4:** Lymph node metastasis rates by tumor size and other factors.

Differentiated versus undifferentiated by size		
Tumor size (cm)	D‐type LNM rate (%)	UD‐type LNM rate (%)
≤ 2	0.8 (3/367)	2.7 (19/693)
2.1–3	3.0 (6/203)	5.1 (15/293)
> 3	3.8 (12/312)	6.6 (24/364)

Ulceration by size		
Tumor size (cm)	LNM rate (%); Ulcer (−)	LNM rate (%); Ulcer (+)
≤ 2	1.7 (16/931)	5.6 (6/113)
2.1–3	4.4 (18/409)	4.5 (3/66)
> 3	5.2 (27/524)	7.8 (9/125)

Lymphovascular Invasion by size		
Tumor size (cm)	LNM rate (%); LVI (−)	LNM rate (%); LVI (+)
≤ 2	1.9 (20/1049)	18.2 (2/11)
2.1–3	3.7 (18/484)	25.0 (3/12)
> 3	4.3 (28/649)	29.6 (8/27)

LNM risk of Undifferentiated, no ulcer and no LVI	
Tumor size (cm)	LNM rate (%); UD‐type UL0 LVI (−) (*n* = 1139)
≤ 1	0 (0/181)
1.1–2	2.5 (11/442)
2.1–3	4.9 (12/245)
> 3	5.5 (15/271)

Abbreviations: D‐type, differentiated‐type; LNM, lymph node metastasis; LVI, lymphovascular invasion; UD‐type, undifferentiated‐type.

Furthermore, mucosal tumors exhibiting ulcers also showed elevated rates of LNM. For smaller tumors, D‐type and UD‐type tumors had metastasis rates of 1.7% and 5.6%, respectively. For medium‐sized tumors, the rates were 4.4% for D‐type and 4.5% for UD‐type. Among large tumors, D‐type had a metastasis rate of 5.2%, while UD‐type was slightly higher at 7.8%. Notably, the presence of an ulcer elevated the risk of LNM, even in smaller tumors.

Notably, tumors associated with LVI had alarmingly high LNM rates. Small, medium, and large‐sized tumors with LVI showed metastasis rates of 18.2%, 25.0%, and 29.6%, respectively. Lastly, in exploring the LNM rates for tumors meeting the expanded criteria for ESD, we assessed UD‐type tumors that lacked ulcers and LVI. UD‐type tumors of 1 cm or less within the ESD criteria demonstrated no cases of lymph node metastasis. Nonetheless, UD‐type tumors measuring 1.1–2 cm under the ESD criteria demonstrate a lymph node metastasis risk of 2.5%. The LMN rates for tumors measuring 2.1–3 cm and greater than 3 cm were 4.9% and 5.5%, respectively.

## 4 Discussion

Our findings provide valuable insights into the risks of LNM in patients with mucosal gastric cancers, focusing particularly on the established ESD criteria. The key findings indicate that LNM occurs at rates of 2%–3% for mucosal cancers meeting the expanded ESD criteria. The scatterplot analysis also predicted that the LNM risk in patients with UD‐type cancers within the ESD criteria might be up to 3.0%. The patterns of LNM analysis showed that LNM occurred in LN station # 1, 3, 4sb, 4d, 5, 6, 7, 8a, 9, 11p. LNM was observed only in the lymph nodes covered by D1 + 11p LN dissection, which suggests that D1 + 11p dissection is sufficient for patients with mucosal gastric cancers.

Consistent with previous findings, the multivariate analysis in this study identified significant risk factors for LNM in patients with EGC. These factors include size, depth of invasion, differentiation type, presence of ulceration, and LVI [8, 9, 23, 24, 25]. However, the reported LNM risks for patients with mucosal gastric cancers satisfying the expanded ESD criteria vary significantly across studies. For instance, multiple large‐scale studies on Korean patients who underwent gastrectomy have reported LNM rates for patients with UD‐type mucosal cancer, without ulceration or LVI and with a tumor size less than or equal to 2 cm, ranging from 1.15%–2.8% [12, 26, 27]. Conversely, data from Western populations suggest even higher risks, with reported LNM rates for patients with EGC within the ESD criteria ranging from 2.9%–14% [15, 28, 29].

The long‐term outcomes of ESD for patients meeting the expanded criteria have shown mixed results. The Japan Clinical Oncology Group (JCOG) trials (JCOG0607 and JCOG1009/1010) demonstrated excellent overall survival for patients undergoing ESD under the expanded criteria, with additional surgery offered to those with incomplete resection or recurrence [30, 31]. These findings laid the foundation for the current Japanese guidelines, highlighting the potential of ESD as a curative treatment for carefully selected patients. However, some studies have reported higher local recurrence rates in the ESD group compared to the gastrectomy group for patients with mucosal gastric cancer. Meta‐analyses have shown significantly higher recurrence‐free survival rates in the surgery group [32, 33]. A recent nationwide multicenter study in Korea found a recurrence rate of 6.7% in patients treated with ESD, with no recurrences in the gastrectomy group [34]. A prospective multicenter cohort study conducted in Korea also demonstrated high overall survival and disease‐specific survival rates for patients who underwent ESD [35]. However, this study primarily focused on patients with D‐type tumors, limited to those with tumors of 3 cm in size or smaller and without ulceration, which did not fully represent the broader patient population eligible for expanded ESD criteria. These findings highlight the curative potential of ESD for carefully selected patients. However, the limited inclusion criteria in some studies and variations in outcomes between different populations suggest the need for further investigation to ensure the broader applicability and safety of the expanded criteria.

One key factor that may influence the observed differences in LNM rates in patients with mucosal cancers is the variation in pathological reporting practices between the JGCA and AJCC/WHO guidelines. In Japan, mucosal gastric cancers are diagnosed based on structural or cytological atypia, not stromal invasion [16, 36]. If a tumor is restricted to the epithelium but has notable structural or cytological atypia, it is diagnosed as an adenocarcinoma. However, the AJCC (8th edition) has a separate category, Tis, for intraepithelial tumors. What is typically classified as “high‐grade dysplasia” is considered mucosal cancer in Japan [37, 38]. Moreover, the presence of tumor emboli in the lymphatic or vascular space is considered when determining the depth of invasion [38]. For example, in Japan, a tumor that directly invades the mucosa but has tumor emboli in the lymphovascular space in the submucosal layer is classified as submucosal carcinoma. In Japan, the population of patients with mucosal gastric cancer includes high‐grade patients with dysplasia and excludes patients with mucosal cancer with LVI at deeper layers. Therefore, patients with mucosal gastric cancer in Korea and other countries may include more advanced staged patients than those in Japan, and logically, patients with mucosal gastric cancer in other countries tend to exhibit a higher incidence of LNM. Because the expanded ESD criteria were originally developed and validated within the Japanese pathological framework, these diagnostic differences limit their direct generalizability to other clinical settings. As a result, applying the expanded criteria without adjustment in non‐Japanese populations may include tumors with a higher intrinsic risk of lymph node metastasis.

An interesting finding from the results is that UD‐type tumors measuring 1 cm or less within the ESD criteria showed no cases of LNM, in contrast to UD‐type tumors with size between 1.1 cm and 2 cm, which showed 2.5% LNM. While the expanded ESD criteria currently permit the resection of UD‐type tumors up to 2 cm, our findings suggest that a stricter threshold of ≤ 1 cm may enhance oncologic safety by minimizing LNM risk. This highlights the need for careful patient selection when considering ESD, particularly for younger patients with a longer life expectancy, where even a small risk of recurrence may be clinically significant. Given these considerations, the decision between ESD and gastrectomy should also account for treatment‐related risks beyond oncologic outcomes. While gastrectomy provides a more definitive lymph node clearance, concerns about surgical morbidity must be addressed. Notably, the mortality rate of patients with EGC undergoing gastrectomy in Korea is relatively low. In 2019, a nationwide survey reported a postoperative mortality rate of 1.0% following gastrectomy for all stages [39]. Various randomized controlled studies of patients with EGC undergoing gastrectomy have shown mortality rates ranging from 0%–0.6% [40, 41, 42]. From a clinical perspective, a 2%–3% risk of occult lymph node metastasis should be carefully weighed against the low operative mortality of gastrectomy in Korea, particularly in fit patients. This trade‐off underscores the need for individualized, risk‐adaptive decision making rather than uniform application of the expanded ESD criteria. Treatment decisions should incorporate both oncologic risk and procedural risk, and a shared decision‐making approach is essential to optimize patient outcomes.

This study has a few limitations that should be acknowledged. First, as a single‐center retrospective study, the generalizability of the findings could be limited. Second, there is a methodological difference in the sectioning width used in this study (4 mm intervals) compared to ESD specimens (2 mm intervals), which could potentially affect the detection of key histological features and LNM, leading to underestimation in surgical cohorts. Third, many patients with mucosal gastric cancer may have undergone ESD as the initial treatment, which introduces a selection bias, as the study cohort primarily includes patients who underwent gastrectomy. Patients with mucosal cancers with large tumor sizes are typically referred to gastrectomy rather than ESD, even though they satisfy the extended ESD criteria. The difference between the patient cohorts between ESD and gastrectomy patients could attribute to higher LNM in gastrectomy patients. Finally, the lack of prospective validation limits the generalizability of our findings, underscoring the need for further multicenter, prospective studies to confirm these results. Notably, outside of Japan, no multicenter, prospective study has been conducted in other regions that has fully validated the extended criteria, emphasizing the need for further research to confirm the broader applicability of these guidelines.

This study provides valuable insights into the risks of LNM in patients with mucosal gastric cancer, particularly in the context of the expanded ESD criteria. ESD remains a promising minimally invasive option; however, the results highlight that LNM risk can still be significant, particularly in cases with larger tumor sizes, ulceration, and LVI. Additionally, differences in pathological reporting between Japan and other countries underscore the importance of regional considerations when applying the ESD guidelines. Accordingly, while the expanded ESD criteria appear appropriate within the Japanese context in which they were developed and validated, their uncritical application in other settings should be approached with caution. In such settings, the classic ESD indications may represent a more conservative oncologic boundary. Our findings emphasize the need for individualized treatment strategies, with thorough patient counseling, particularly for younger patients with good performance status. Treatment decisions should therefore be context‐dependent, based on pathological framework, patient characteristics, and risk tolerance. Further multicenter, prospective studies are warranted to validate and refine these criteria across different clinical and pathological settings.

## Funding

This study was supported by philanthropic donations from individual patients made to the Seoul National University Hospital research fund (institutional project number: 3020240040).

## Ethics Statement

This study was approved by the Institutional Review Board of Seoul National University Hospital (No. 2311‐075‐1483).

## Conflicts of Interest

The authors declare no conflicts of interest.

## Data Availability

The data that support the findings of this study are available on request from the corresponding author. The data are not publicly available due to privacy or ethical restrictions.
